# Safety of RTS,S/AS01E vaccine for malaria in African children aged 5 to 17 months: A systematic review and meta-analysis of randomized controlled trials

**DOI:** 10.1371/journal.pgph.0004387

**Published:** 2025-06-16

**Authors:** Joseph Alexis Zoa, Rahi-metou Njemguie Linjouom, Martin Nyangono Ndongo, Jan Rene Nkeck

**Affiliations:** 1 Department of Pharmaceutical Sciences, Faculty of Medicine and Pharmaceutical Sciences, The University of Ebolowa, Sangmelima, Cameroon; 2 Department of Galenics and Pharmaceutical Legislation, Faculty of Medicine and Biomedical Sciences, University of Yaoundé I, Yaoundé, Cameroon; 3 Department of Internal Medicine and Specialities, Faculty of Medicine and Biomedical Sciences, The University of Yaoundé I, Yaoundé, Cameroon; Malaria Research and Training Center, University of Science, Techniques and Technology of Bamako, MALI

## Abstract

**Background:**

Numerous randomized controlled trials (RCTs) have demonstrated the safety of the RTS,S/AS01E vaccine in African children aged 5–17 months. However, conflicting evidence surrounding serious adverse events (SAEs) has prompted calls for further research to determine whether the benefits of the vaccine outweigh its risks, particularly in high-risk populations such as young children in malaria-endemic regions.

**Methods:**

On February 12, 2025, a comprehensive literature search was performed across PubMed, Wiley Online Library, and Web of Science. The Patient/Population, Intervention, Comparison, Outcomes, and Study Design (PICOS) framework was employed to screen and select relevant studies. Following initial screening, titles, abstracts, and full texts were filtered using EndNote software. Data were systematically extracted from each included study. SAEs were categorized according to the Medical Dictionary for Regulatory Activities (MedDRA) terminology. The analysis focused on calculating proportions (%), risk ratios (RRs), and odds ratios (ORs) of SAEs. Pooled effect sizes and 95% confidence intervals (CIs) were derived using random-effects models from aggregated data meta-analysis. Publication bias was evaluated using the Begg’s test, and Egger’s test, with statistical significance assessed via Z tests.

**Results:**

This systematic review and meta-analysis included 11 studies involving 27949 children (16976 vaccinated with RTS,S/AS01E and 10973 vaccinated with rabies vaccine). The overall proportion of SAEs was 21.4% [95% CI: 17.5–25.5] in the RTS,S/AS01E group, compared to 26.2% [95% CI: 22.4–30.2] in the control group (rabies vaccine), yielding a pooled RR of 0.80 [95% CI: 0.72–0.88, *P* < 0.0001]. The pooled frequency of death was 1.37% [95% CI: 0.53-2.61] in the RTS,S/AS01E group and 1,31% [95% CI: 0.49-2.52] in the control group, with a non-significant odds ratio (OR) of 1.08% [95% CI: 0.83-1.41, *P* = 0.56]. Common SAEs included febrile convulsions, pneumonia, and severe malaria, with severe malaria being significantly more prevalent in children who received the rabies vaccine [OR = 0.61, 95% CI: 0.53-1.26, *P* < 0.00001]. The overall methodological quality of the included studies was high, with a low probability of bias.

**Conclusions:**

The risk of SAEs is comparable between children who received the RTS,S/AS01E vaccine and those who received the rabies vaccine, except for severe malaria, which is significantly more frequent in the rabies vaccine group. This suggests that the RTS,S/AS01E vaccine may provide meaningful protection against severe forms of malaria, a critical outcome in high-burden regions. However, further long-term data are needed to confirm these findings and address several unresolved questions.

**PROSPERO registration number:**

CRD42024321008.

## 1. Introduction

Malaria remains a significant global health challenge, with the World Health Organization (WHO) reporting over 249 million cases [1]. The proportion of total malaria deaths among children under 5 years of age decreased from 86.7% in 2000 to 73.7% in 2023 [2]. *Plasmodium falciparum* (Pf) and *Plasmodium vivax* (Pv) are the primary species responsible for the global malaria burden, with *Pf* being the predominant species in sub-Saharan Africa [3,4]. While vector control and chemoprevention have reduced mortality, emerging artemisinin resistance and insecticide-resistant mosquitoes threaten progress [5–8], necessitating complementary tools like vaccines [9]. In 2021, WHO endorsed two vaccines: RTS,S/AS01E, the first malaria vaccine to complete Phase 3 trials, and R21/Matrix-M, a higher-volume, lower-cost alternative [10,11]. By 2024, over 20 African countries had integrated these into immunization programs, with real-world evidence showing 13% reductions in child mortality (RTS,S) and 75–80% seasonal efficacy (R21) [2,11–13].

The RTS,S/AS01E vaccine, despite its benefits, has raised safety considerations. Phase 3 trials and the Malaria Vaccine Implementation Programme (MVIP) identified a transient increase in febrile convulsions post-vaccination, without long-term sequelae [10,14]. Gender-specific analyses noted a non-significant mortality trend in girls [15,16], though subsequent phase IV trials and MVIP data confirmed its net mortality benefit [13,17]. WHO now recommends fever management protocols and gender-disaggregated monitoring to mitigate risks [2,18,19]. However, inconsistent safety data persist, particularly regarding dose-dependent reactions such as febrile seizures and sex-disparate outcomes [15,16]. While some studies report higher all-cause mortality in vaccinated girls [20], others found no significant association [17], highlighting the need for rigorous demographic analyses. The phase IV study by Asante et al. (2024), conducted among 652673 children in Ghana, Kenya, and Mali, found that the introduction of the RTS,S vaccine had a comparable impact on mortality in both girls and boys (relative mortality ratio: 1.03 [95% CI 0.88–1.21]) [13].

Although public demand for the vaccine remains strong [21], persistent safety concerns continue to erode confidence. This systematic review and meta-analysis evaluates the RTS,S/AS01E vaccine’s safety profile through a transparent assessment of RCTs data. By synthesizing evidence from clinical and implementation phases, this study aims to clarify the risk-benefit profile of RTS,S/AS01E, informing policy decisions and ensuring safe, equitable deployment in endemic regions.

## 2. Materials and methods

This systematic review and meta-analysis followed the preferred reporting items for systematic reviews and meta-analyses (PRISMA) reporting guideline [22]. The study protocol is registered with the protocol ID CRD42024321008 available from https://www.crd.york.ac.uk/prospero/display_record.php?ID=CRD42024321008.

### 2.1 Search strategy

We performed a literature search on February 12, 2025, utilizing the PubMed, Wiley Online Library (WOL), and Web of Science (WS) databases regardless of publication language. Additional relevant records were identified by reading the full texts and reviewing the reference lists of the retrieved articles. The detailed search methodology is provided in the Table 1.

**Table 1:** Literature search strategy for Pubmed, Web of Science and Wiley online library databases.

SEARCH TERMS	RESULTS		
	Pubmed	Web of science	Wiley online library
#1 “RTS,S/AS01” OR “RTSS/AS01”	292	108	118
#2 “safety” OR “serious adverse events” OR “SAE” OR “innocuity” OR “severe effects” OR “complications”	4,312,598	1,101,203	2,403,540
#1AND#2	70	41	96

Search update: 12/02/2025

### 2.2 Study selection

The authors, Zoa and Njemguie, performed a standardized, unblinded screening of potentially relevant articles. The process began with an evaluation of titles and abstracts, followed by full-text reviews when necessary to assess eligibility based on the predefined inclusion and exclusion criteria. Any discrepancies between the reviewers regarding study selection were resolved through discussion and consensus, with a third author (Nkeck) consulted if needed to make a final decision.

### 2.3 Inclusion criteria

The criteria for eligible studies included the following:

**(P) Population**: Children aged 5–17 months at the time of the first vaccination and residing permanently in malaria-endemic areas in Sub-Saharan Africa.
**(I) Intervention**: RTS,S/AS01E vaccine according to the 0–1–2 and 0–1–2–20 months vaccination schedule.
**(C) Comparison**: Rabies vaccine or Hepatitis A vaccine
**(O) Outcomes**: The proportions of any SAE. The proportion was defined as the ratio of the number of individuals reporting at least one SAE to the total number of individuals in a given group throughout the study period. SAE refer to significant, severe or life-threatening medical occurrences or conditions that participants may experience after receiving the vaccine. These events go beyond the expected side effects and pose a substantial risk to the health and well-being of the individuals involved [23]. SAEs were classified according to the terminology provided by the Medical Dictionary for Regulatory Activities (MedDRA) [24].
**(S) Study design**: Randomized controlled/clinical trials (RCT).

### 2.4 Exclusion criteria

Using EndNote software, Zoa and Njemguie applied the exclusion criteria. Exclusions were based on the following: (I) duplicate publications; (II) Non-randomized clinical trials (conference papers, letters, reviews, case reports, communications, expert opinions, basic research, or cohort studies); (III) studies unrelated to the RTS,S/AS01E vaccine; (IV) studies that did not focus on the 5–17 months age group; and (V) studies lacking reported proportions of SAEs. Any disagreements between the reviewers regarding study eligibility were resolved through discussion or, if necessary, by consulting a third author, Nkeck, to reach a consensus.

### 2.5 Data extraction

The authors, Nyangono and Nkeck, independently extracted data from studies that met the inclusion criteria. The extracted data included: (I) study ID, (II) country, (III) follow-up duration (in months) and type of follow-up (passive or active), (IV) study design, (V) sample size, (VI) schedule, (VII) the number of participants, and (VIII) the proportion of SAEs in both the RTS,S/AS01E and control groups.

### 2.6 Assessment of the risk of bias in the included studies

The authors, Zoa and Nyangono, evaluated the quality of the literature included in this study using the “risk of bias assessment” tool from the Cochrane systematic review [25]. The assessment criteria encompass the following seven areas: generation of randomized sequences, allocation concealment, blinding of investigators and subjects, blinding of outcome evaluators, completeness of outcome information, selective reporting of study results, and other sources of bias. Each item can be described as “low risk of bias”, “high risk of bias”, or “unclear risk”. Eventually, the evaluations mentioned above were combined to create a risk of bias assessment map and quality outcomes for each piece of literature. If there is a disagreement between the two researchers, a third researcher (Nkeck) conducted an independent assessment, and the final assessment was determined through mutual agreement among the three researchers.

### 2.7 Data analysis

The Mantel-Haenszel method was used for the meta-analysis of relative risk (RR) and odds ratio (OR). RR is a measure that compares the risk of experiencing SAE between malaria vaccine and control groups, while OR is a measure that compares the odds of a specific SAE between malaria vaccine and control groups [26]. For rare outcomes with prevalence less than 10% such as death, we reported the odds ratio (OR), which serves as an approximation of the RR [27]. OR greater than 1 indicates that there is a higher likelihood or increased odds of a specific SAE in the RTS,S/AS01E group compared to the control group. The p-values for RR and OR were derived using the Z test, with a P < 0.05 considered statistically significant. Between-study heterogeneity was assessed using Cochran’s Q test, and the magnitude of heterogeneity was quantified using the I² statistic [28]. In the cases of significant heterogeneity (I^2^ > 50% or P < 0.05), the random effects model was used for the meta-analysis. Otherwise, a fixed-effects model was adopted. The potential for publication bias was investigated using inspection of the Begg’s funnel plot and Egger’s linear regression with P < 0.1 defining publication bias [29]. We conducted subgroup analyses according to RCT phases (II and III). The meta-analyses were performed using aggregated data with Review Manager (RevMan) version 5.4.1 (The Cochrane Collaboration, 2020) and MedCalc 20.1.4. All statistical tests were two-sided.

## 3. Results

### 3.1 Study selection and characteristics

**Fig 1:**
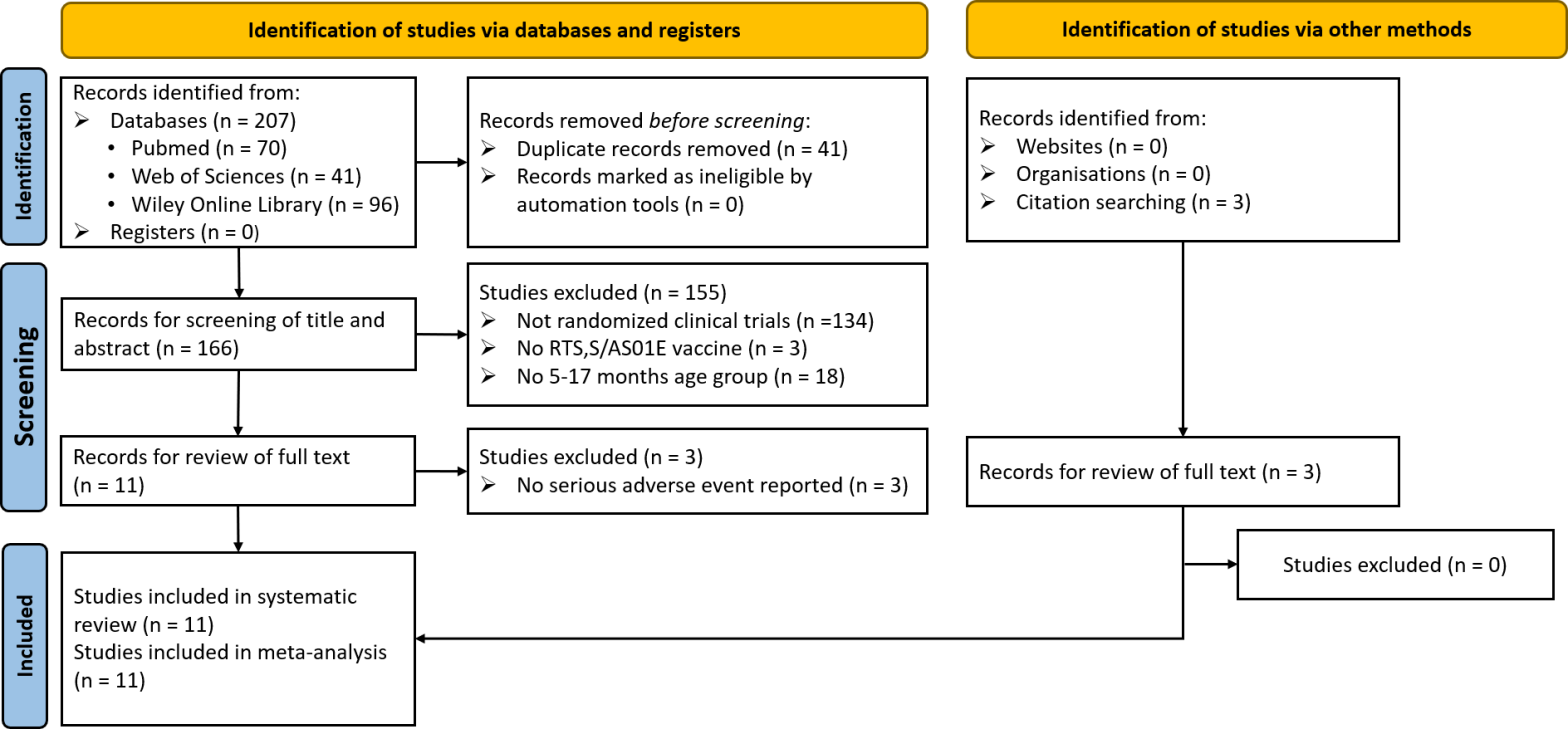
Study flow diagram.

A flow diagram illustrating our literature search and screening process is provided in Fig 1. Initially, we identified 207 records from databases. After removing 41 duplicates, we screened 166 records by reviewing their titles and abstracts. We excluded 134 studies unrelated to randomized controlled trials (RCTs), 3 studies unrelated to the RTS,S/AS01E vaccine, and 18 studies that did not include the 5–17 months age group. Subsequently, we assessed 14 full-text articles for eligibility (12 from databases and 3 from citation searches). Of these, we excluded 3 full-text articles because they did not report SAEs. Ultimately, we included 11 articles involving 27949 children (16976 recipients of RTS,S/AS01E and 10973 control recipients) in the meta-analysis [16,30–39]. A summary of all records identified in the literature search, including those that were excluded from the analyses is presented in supporting information (S1 Table).

The included studies were published between 2008 and 2022. 4907 of 27949 (17.56%) participants were from Kenya, 4542 (16.25%) from Ghana, 4418 (15.81%) from Tanzania, 3600 (12.88%) from Gabon, 3494 (12.5%) from Mozambique, 3494 (12.5%) from Burkina-Faso and 3494 (12.5%) from Malawi. All 11 studies used the rabies vaccine as the control [16,30–39]. Among these, nine provided data on the number of child deaths during the study period [16,30–32,34,35,37–39], while six reported the incidence of febrile convulsions [16,32,34–36,38]. Severe malaria cases were documented in six studies [31,32,35–37,39], and pneumonia cases were reported in five [30,32,35–37]. Additional details on these eligible studies are presented in Table 2.

**Table 2:** Extracted data of the Included Randomized Controlled Clinical Trial Studies on the Safety of RTS,S/AS01E.

Nº	Study ID	Country	Follow-up time (months)	Follow-up type	Phase of RCT	Sample size	Control	Schedule			
1	Bejon, 2008 [30]	Kenya, Tanzania	7.9	Active	IIB	894	Rabies vaccine	3 doses (0, 1, 2)	**Serious adverse events (SAE)**	**RTS,S/ AS01E** (N = 447)	**Control** (N = 447)
									All (%)	10.5	18.3
									Fatal or death	1	1
									Pneumonia	18	26
									Gastro-enteritis	10	21
2	Owusu-Agyei, 2009 [31]	Ghana	19	Active	II	135	Rabies vaccine	3 doses (0, 1, 2)	**Serious adverse events (SAE)**	**RTS,S/ AS01E** (N = 90)	**Control** (N = 45)
									All (%)	14.4	13.3
									Fatal or death	0	1
									Severe malaria	0	1
3	Lusingu, 2010 [32]	Kenya, Tanzania	14	Active	IIB	894	Rabies vaccine	3 doses (0, 1, 2)	**Serious adverse events (SAE)**	**RTS,S/ AS01E** (N = 447)	**Control** (N = 447)
									All (%)	11.4	19.7
									Fatal or death	1	0
									Severe malaria	9	31
									Pneumonia	16	26
									Febrile convulsion	14	20
									Anemia (:)	5	2
									Gastroenteritis	10	22
4	Olotu, 2011 [33]	Ghana, Tanzania	18	Active	IIB	894	Rabies vaccine	3 doses (0, 1, 2)	**Serious adverse events (SAE)**	**RTS,S/ AS01E** (N = 447)	**Control** (N = 447)
									All (%)	11.4	19.7
5	Agnandji, 2011 [34]	Burkina Faso, Gabon, Ghana, Kenya, Malawi, Mozambique, Tanzania	18	Passive	III	8923	Rabies vaccine	4 doses (0, 1, 2, 20)	**Serious adverse events (SAE)**	**RTS,S/ AS01E** (N = 5949)	**Control** (N = 2974)
									All (%)	17.6	21.6
									Fatal or death	56	28
									Febrile convulsion	211	106
									Meningitis	11	1
6	Minsoko, 2014 [35]	Burkina Faso, Gabon, Ghana, Kenya, Malawi, Mozambique, Tanzania	20	Passive	III	8923	Rabies vaccine	4 doses (0, 1, 2, 20)	**Serious adverse events (SAE)**	**RTS,S/ AS01E** (N = 5949)	**Control** (N = 2974)
									All (%)	18.6	22.7
									Fatal or death	4	4
									Severe malaria	400	313
									Febrile Convulsion	224	112
									Meningitis	9	1
									Pneumonia	353	189
									Anemia	190	155
									Enteritis	21	12
7	Olotu, 2016 [36]	Kenya	84	Passive	II	447	Rabies vaccine	3 doses (0, 1, 2)	**Serious adverse events (SAE)**	**RTS,S/ AS01E** (N = 223)	**Control** (N = 224)
									All (%)	17.9	25.3
									Severe malaria	14	30
									Febrile convulsions	15	20
									Pneumonia	9	10
									Anemia	5	12
									Gastroenteritis	7	12
8	Otieno, 2016 [38]	Kenya	14	Passive	III	200	Rabies vaccine	3 doses (0, 1, 2)	**Serious adverse events (SAE)**	**RTS,S/ AS01E** (N = 99)	**Control** (N = 101)
									All (%)	41.4	36.6
									Fatal or Death	5	4
									Febrile convulsions	10	13
									Malnutrition	7	5
9	Mendoza, 2019 [15]	Burkina Faso, Gabon, Ghana, Kenya, Malawi, Mozambique, Tanzania	48	Passive	III	5950	Rabies vaccine	4 doses (0, 1, 2, 20)	**Serious adverse events (SAE)**	**RTS,S/ AS01E** (N = 2976)	**Control** (N = 2974)
									All (%)	24.2	28.4
									Fatal or Death	56	45
									Febrile convulsions	7	2
									Meningitis	11	1
10	Otieno, 2020^*^ [37]	Burkina Faso, Gabon, Ghana, Kenya, Malawi, Mozambique, Tanzania	39	Passive	III	99	Rabies vaccine	4 doses (0, 1, 2, 20)	**Serious adverse events (SAE)**	**RTS,S/ AS01E** (N = 51)	**Control** (N = 48)
									All (%)	92.2	87.5
									Fatal or Death	15	15
									Pneumonia	14	22
									Severe malaria	7	10
11	Samuels, 2022 [39]	Ghana, Kenya	21	Passive	IIb	591	Rabies vaccine	4 doses (0, 1, 2, 20)	**Serious adverse events (SAE)**	**RTS,S/ AS01E** (N = 298)	**Control** (N = 293)
									All (%)	16	24
									Fatal or Death	1	0
									Severe malaria	13	31

* WHO Stage 1,2 HIV disease, RCT = Randomized controlled trial

### 3.2 Meta-analysis

Eleven studies provided data to estimate the pooled proportion of all SAEs and their relative risks (RR) [16,30–39].

#### 3.2.1 Risk of SAE in RTS,S/AS01E and control groups.

The pooled proportion of SAEs among children who received RTS,S/AS01E was 21.4% (11 studies, 16976 children, 95% CI [17.5–25.5]), and 26.2% (11 studies, 10973 children, 95% CI [22.4-30.2]) in the control group as shown in Table 3.

**Table 3:** Sample size, proportion and corresponding 95% CIs of the SAEs in RTS,S/AS01E and Control groups.

Groups	Parameters	Serious adverse events (SAEs)				
		Overall SAEs	Fatal SAE or death	Severe malaria	Febrile convulsions	Pneumonia
**RTS,S/AS01E**	Sample size	16976	16306	1638	10619	2093
	Proportion	21.4%	1.37%	14.8%	8.79%	15.7%
	95% CI	17.5–25.5	0.53-2.61	2.7-34.1	2,97-17,28	3.4-34.5
**Control**	Sample size	10973	10303	1207	7229	1675
	Proportion	26.2%	1.31%	27.8%	8.82%	18.1%
	95% CI	22.4-30,2	0.49-2.52	10.5-45.1	2.93-17.45	6.9-33.1

Proportions were calculated according to the random effects model. Medcalc 20.1.4; CI: confidence interval

#### 3.2.2 Comparison of the risk of overall SAE in children receiving RTS,S/AS01E and controls.

**Fig 2:**
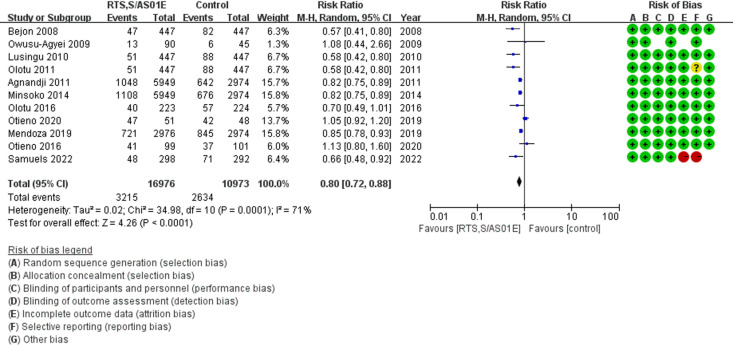
Forest plot of comparison: overall serious adverse events [RTS,S/AS01E vs Control] in 5-17 months children, outcome: Risk Ratio (RR).

We used a random effect model to calculate the RR and 95% CI for SAE due to statistical heterogeneity (I^2^ = 71%). The findings revealed that the children in RTS,S/AS01E group had a significant reduced risk of SAE (11 studies, 27949 children, RR = 0.80, 95% CI [0.72, 0.88], *P *< 0.0001, Fig 2)

#### 3.2.3 Comparison of the risk of the frequently reported SAE in children receiving RTS,S/AS01E and controls.

**Fig 3:**
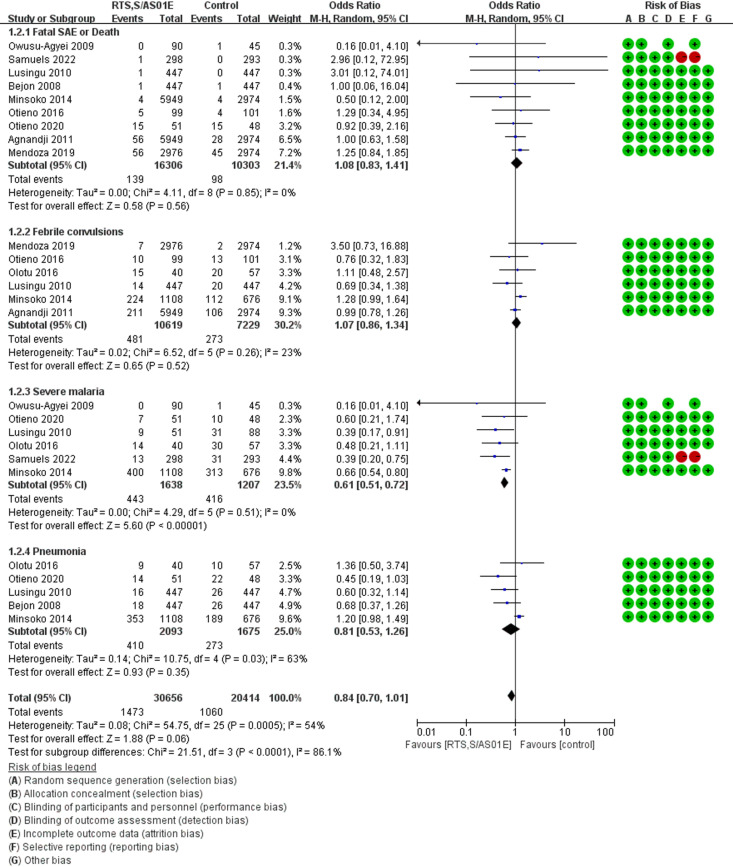
Forest plot of comparison: the frequently reported serious adverse events [RTS,S/AS01E vs Control] in 5-17 months children, outcome: Odds Ratio (OR).

**Risk of fatal SAE or death**: The pooled frequency of deaths in children receiving RTS,S/AS01E was 1.37% (9 studies, 16306 children, 95% CI [0.53-2.61], Table 3), while the pooled frequency in control was 1,31% (9 studies, 10303 children, 95% CI [0.49-2.52]) with a non-significant increased odds for death in RTS,S/AS01E group than in control group (OR = 1.08 [0.83-1.41], *P* = 0.56, Fig 3).

**Risk of febrile convulsions**: The pooled frequency of febrile convulsions in children receiving RTS,S/AS01E was 8,79% (6 studies, 10619 children, 95% CI [2.97-17.28], Table 3), while the pooled frequency in control was 8,82% (6 studies, 7229 children, 95% CI [2.93-17.45]) with a non-significant increased odds for febrile convulsion in RTS,S/AS01E group than in control group (OR = 1.07 [0.86-1.34], *P* = 0.50, Fig 3).

**Risk of pneumonia**: The pooled frequency of pneumonia in children receiving RTS,S/AS01E was 15.7% (4 studies, 2093 children, 95% CI [3.4-34.5]), while the pooled frequency in control was 18.1% (4 studies, 1675 children, 95% CI [6.9-33.1], Table 3) with a non-significant decreased odds for pneumonia in RTS,S/AS01E group than in control group (OR = 0.81 [0.53-1.26], *P* = 0.35, Fig 3).

**Risk of severe malaria**: The pooled frequency of severe malaria in children receiving RTS,S/AS01E was 14.8% (4 studies, 1638 children, 95% CI [2.7-34.1], Table 3), while the pooled frequency in control was 25.8% (4 studies, 1207 children 95% CI [10.5-45.1]) with a significant decreased odds for severe malaria in RTS,S/AS01E group than in control group (OR = 0.61 [0.51-0.72], *P* < 0.00001, Fig 3).

#### 3.2.4 Sensitivity analysis.

**Fig 4:**
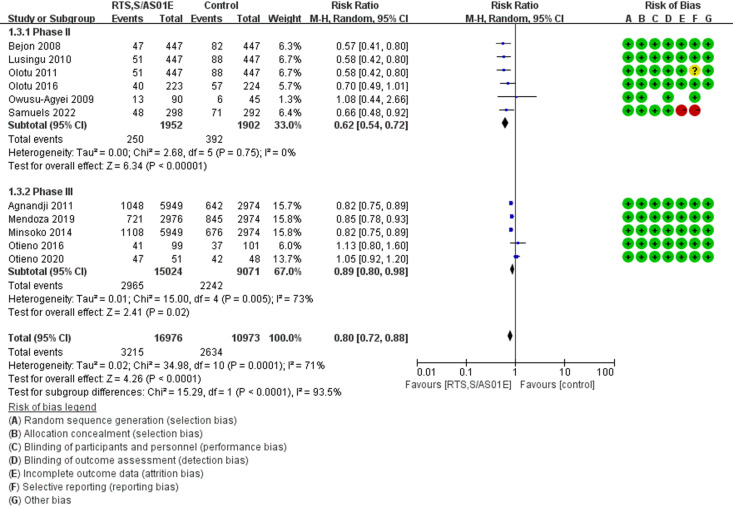
Forest plot of comparison: Subgroup analysis [RTS,S/AS01E vs Control] in 5-17 months children, outcome: Risk Ratio (RR).

Due to significant heterogeneity observed in the data, we conducted a subgroup analysis stratified by study phase. The analysis revealed that children receiving the RTS,S/AS01E vaccine had a significantly reduced risk of SAEs compared to the control group. In phase 2 trials (6 studies, 3854 children), the relative risk (RR) was 0.62 (95% CI [0.54-0.72], *P* < 0.00001) [30–33,36,39]. Similarly, in phase 3 trials (5 studies, 24095 children), the relative risk was 0.89 (95% CI [0.80-0.98], *P* = 0.02) [16,34,35,37,38]. These findings suggest a consistent protective effect of the vaccine across both phases, though the magnitude of risk reduction varied. The results of the sensitivity analysis are presented in Fig 4.

### 3.3 Evaluation of publication bias

**Fig 5:**
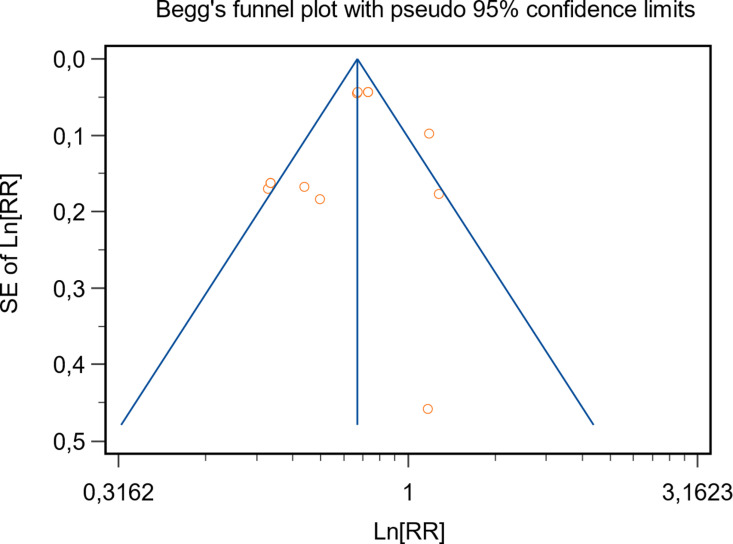
Risk of bias graph: Begg’s funnel plot. RR, risk ratio; SE, standard error of RR.

To ensure the reliability of our meta-analysis, we rigorously assessed the risk of publication bias and the methodological quality of the included studies. Using Egger’s test, we found no significant evidence of publication bias (P = 0.40; 95% CI: -2.73-1.22; Fig 5), indicating that the findings are unlikely to be influenced by selective reporting.

A thorough quality assessment of the included studies revealed that the majority demonstrated low risks of bias across key domains, including selection, performance, detection, and reporting. This high methodological quality can be attributed to the rigorous design and execution of the RCTs [17,32,33,35–37,40,41], which featured proper randomization processes, adherence to intended interventions, accurate outcome measurement, minimal missing data, and transparent reporting of results (Table 4). However, one study was identified as having a high risk of bias in selective reporting of study and completeness of outcome information [39].

**Table 4:** Risk of bias and quality/certainty assessments for each study according to the Cochrane risk-of-bias tool for randomized trials.

Nº	Study ID	Generation of randomized sequences	Allocation concealment	Blinding of investigators and subjects	Blinding of outcome evaluators	Completeness of outcome information	Selective reporting of study results	Other sources of bias	Over-all
1	Bejon, 2008	+	+	+	+	+	+	+	Low
2	Owusu-Agyei, 2009	+	+	?	+	?	+	?	Unclear
3	Lusingu, 2010	+	+	+	+	+	+	+	Low
4	Olotu, 2011	+	+	+	+	+	+	+	Low
5	Agnandji, 2011	+	+	+	+	+	+	+	Low
6	Minsoko, 2014	+	+	+	+	+	+	+	Low
7	Olotu, 2016	+	+	+	+	?	+	+	Low
8	Otieno, 2016	+	+	+	+	+	+	+	Low
9	Mendoza, 2019	+	+	+	+	+	+	+	Low
10	Otieno, 2019	+	+	+	+	+	+	+	Low
11	Samuels, 2022	+	+	+	+	–	–	?	High

+ = Low risk;? = unclear risk; - = high risk

In conclusion, the overall methodological quality of the included studies was high, with a low probability of bias. This strengthens the validity of our meta-analysis and supports the robustness of its findings.

## 4. Discussion

The RTS,S/AS01E malaria vaccine is a landmark achievement in combating malaria, particularly among children in endemic regions. The RTS,S/AS01E vaccine’s journey began with phase 2 trials conducted before 2021, targeting children aged 5–17 months. These trials established a favorable benefit-risk ratio, despite transient reactogenicity such as fever [30–33,36,39]. A slight increase in febrile convulsions was noted, prompting further investigation, but no long-term neurologic sequelae were observed, laying a solid foundation for the vaccine’s safety profile. By 2021, phase 3 trials expanded on earlier findings, incorporating rigorous monitoring of febrile convulsions within 7 days post-vaccination, following the Brighton Collaboration Working Group’s case definition. These trials reported a non-significant increase in febrile convulsions, no significant differences in mortality or pneumonia, and a 31% reduction in severe malaria [16,34,35,37,38]. The attenuated protective effect compared to phase 2 (RR = 0.62) likely reflects variations in trial design or malaria endemicity, reinforcing the vaccine’s efficacy in high-transmission settings [40,41]. In 2021, Chandramohan et al. conducted a pivotal trial in seasonal malaria settings, identifying a transient febrile seizure risk (7.1 vs. 2.3/1,000 doses) [17], consistent with phase 3 findings [16,34,35,37,38]. Although excluded from our meta-analysis, their conclusion that this risk is manageable with fever monitoring, aligned with phase 3 data and supported the WHO recommendations [2,10,42]. The absence of long-term neurologic sequelae further bolstered confidence in the vaccine’s safety.

Our meta-analysis synthesized data to evaluate RTS,S/AS01E’s safety comprehensively. The analysis found a significantly lower SAEs in the vaccine group and confirmed a reduction in severe malaria. No significant differences were observed in mortality, pneumonia and febrile convulsions, reinforcing the vaccine’s suitability for large-scale use [43–45]. In 2024, Asante et al.’s real-world study reported a 32% reduction in severe malaria admissions and a 9% reduction in all-cause mortality (excluding injury) [13]. Our metanalysis may miss long-term mortality benefits seen in real-world settings; RCTs are underpowered for rare outcomes. Asante et al.’s findings reflect real-world population-level benefits, potentially influenced by healthcare access and pandemic-related shifts in health-seeking behavior, such as reduced hospitalizations due to fear of infection. Their higher death rate may reflect a wider age range (1–59 months). Both studies agreed on no vaccine-attributable mortality and no excess non-malaria infections (e.g., pneumonia), solidifying the vaccine’s safety in programmatic settings [13,46].

Key limitations of our meta-analysis include the reliance on aggregated data, lacking individual participant data (IPD) to account for multiple SAEs or person-time. Heterogeneity in SAE definitions (I² = 66%) and underrepresentation of high-risk groups, such as HIV-exposed children or infants under 5 months, were noted across studies. Geographic imbalances (e.g., only 16.25% of participants from West Africa) and short follow-up periods (≤48 months) further limit generalizability. Despite vaccine hesitancy fueled by safety concerns and misinformation [47], our findings confirm RTS,S/AS01E’s strong safety record. From phase 2 trials to Asante’s 2024 study, RTS,S/AS01E has proven its value in malaria control [13,17,46]. The consistent reduction in severe malaria, manageable febrile seizure risk, and absence of mortality signals support its integration into comprehensive strategies. Addressing hesitancy and research gaps will maximize its impact, advancing the goal of malaria elimination.

## 5. Conclusion

The RTS,S/AS01E malaria vaccine represents a major advancement in the fight against malaria, particularly for children in high-transmission regions. Extensive clinical trials and real-world evidence have demonstrated its favorable benefit-risk profile, marked by a significant reduction in severe malaria and manageable, transient safety concerns such as febrile convulsions, with no evidence of long-term neurological complications or vaccine-attributable mortality. While vaccine hesitancy fueled by misinformation remains a challenge, the data robustly support its safety and efficacy, aligning with WHO recommendations for integration with existing malaria control measures.

## Supporting information

S1 Table numbered table of all studies identified in the literature search, including those that were excluded from the analyses. (DOCX)

S1 File Endnote library for our manuscript. (ENL)
