# Supplementary material for: Safety of RTS,S/AS01E vaccine for malaria in African children aged 5 to 17 months: A systematic review and meta-analysis of randomized controlled trials
Source: PLOS Glob Public Health. 2025 Jun 16;5(6):e0004387. doi: 10.1371/journal.pgph.0004387 (PMC12169556; doi:10.1371/journal.pgph.0004387)
Supplement: S1 Table — (DOCX) [file pgph.0004387.s001.docx]

S1 Table: Summary of all records identified in the literature search, including those that were excluded from the analyses.

| **EXCLUDED STUDIES** | | | | |
| --- | --- | --- | --- | --- |
| **N°** | **Study ID** | **Year** | **Screening** | **Reason of exclusion** |
| 1 | Animal Cell Technology. Advanced Fermentation and Cell Technology2021. p. 579-685. | 2001 | title/abstract | Not randomized clinical trial |
| 2 | Lell B, Agnandji S, von Glasenapp I, Haertle S, Oyakhiromen S, Issifou S, et al. A randomized trial assessing the safety and immunogenicity of AS01 and AS02 adjuvanted RTS,S malaria vaccine candidates in children in Gabon. PloS one. 2009;4(10):e7611. | 2009 | title/abstract | no 5-17 months age group |
| 3 | Moorthy VS, Ballou WR. Immunological mechanisms underlying protection mediated by RTS,S: a review of the available data. Malar J. 2009;8:312. | 2009 | title/abstract | Not randomized clinical trial |
| 4 | Waitumbi JN, Anyona SB, Hunja CW, Kifude CM, Polhemus ME, Walsh DS, et al. Impact of RTS,S/AS02(A) and RTS,S/AS01(B) on genotypes of P. falciparum in adults participating in a malaria vaccine clinical trial. PloS one. 2009;4(11):e7849. | 2009 | title/abstract | no 5-17 months age group |
| 5 | BALLOU WR. The development of the RTS,S malaria vaccine candidate: challenges and lessons. Parasite Immunology. 2009;31(9):492-500. | 2009 | title/abstract | Not randomized clinical trial |
| 6 | Agnandji ST, Asante KP, Lyimo J, Vekemans J, Soulanoudjingar SS, Owusu R, et al. Evaluation of the safety and immunogenicity of the RTS,S/AS01E malaria candidate vaccine when integrated in the expanded program of immunization. J Infect Dis. 2010;202(7):1076-87. | 2010 | title/abstract | no 5-17 months age group |
| 7 | Cohen J, Benns S, Vekemans J, Leach A. [The malaria vaccine candidate RTS,S/AS is in phase III clinical trials]. Ann Pharm Fr. 2010;68(6):370-9. | 2010 | title/abstract | Not randomized clinical trial |
| 8 | Cohen J, Nussenzweig V, Nussenzweig R, Vekemans J, Leach A. From the circumsporozoite protein to the RTS, S/AS candidate vaccine. Hum Vaccin. 2010;6(1):90-6. | 2010 | title/abstract | Not randomized clinical trial |
| 9 | Ansong D, Asante KP, Vekemans J, Owusu SK, Owusu R, Brobby NA, et al. T cell responses to the RTS,S/AS01(E) and RTS,S/AS02(D) malaria candidate vaccines administered according to different schedules to Ghanaian children. PloS one. 2011;6(4):e18891. | 2011 | full text | no SAE reported |
| 10 | Asante KP, Abdulla S, Agnandji S, Lyimo J, Vekemans J, Soulanoudjingar S, et al. Safety and efficacy of the RTS,S/AS01E candidate malaria vaccine given with expanded-programme-on-immunisation vaccines: 19 month follow-up of a randomised, open-label, phase 2 trial. The Lancet Infectious diseases. 2011;11(10):741-9. | 2011 | title/abstract | no 5-17 months age group |
| 11 | Leach A, Vekemans J, Lievens M, Ofori-Anyinam O, Cahill C, Owusu-Agyei S, et al. Design of a phase III multicenter trial to evaluate the efficacy of the RTS,S/AS01 malaria vaccine in children across diverse transmission settings in Africa. Malar J. 2011;10:224. | 2011 | full text | no SAE reported |
| 12 | Olotu A, Moris P, Mwacharo J, Vekemans J, Kimani D, Janssens M, et al. Circumsporozoite-specific T cell responses in children vaccinated with RTS,S/AS01E and protection against P falciparum clinical malaria. PloS one. 2011;6(10):e25786. | 2011 | title/abstract | Not randomized clinical trial |
| 13 | From the Australian Commission on Safety and Quality in Health Care: Preventing medication errors by using Tall Man lettering. Medical Journal of Australia. 2011;195(9):493-. | 2011 | title/abstract | Not randomized clinical trial |
| 14 | Irwin W. Sherman | 2011 | title/abstract | Not randomized clinical trial |
| 15 | WHO malaria policy advisor and secretariat | 2012 | title/abstract | Not randomized clinical trial |
| 16 | Agnandji ST, Lell B, Fernandes JF, Abossolo BP, Methogo BG, Kabwende AL, et al. A phase 3 trial of RTS,S/AS01 malaria vaccine in African infants. N Engl J Med. 2012;367(24):2284-95. | 2012 | title/abstract | Not randomized clinical trial |
| 17 | Cross-cutting topics. Tropical Medicine & International Health. 2012;17(s1):66-80. | 2012 | title/abstract | Not randomized clinical trial |
| 18 | Alan Gunn, Sarah J. Pitt | 2012 | title/abstract | Not randomized clinical trial |
| 19 | Le Grand LR, White M, Siegel EB, Barnard RT. Recombinant Vaccines: Development, Production, and Application. Pharmaceutical Biotechnology2012. p. 423-49. | 2012 | title/abstract | Not randomized clinical trial |
| 20 | Oral Presentations. Tropical Medicine & International Health. 2013;18(s1):52-107. | 2013 | title/abstract | Not randomized clinical trial |
| 21 | Chang CC, Crane M, Zhou J, Mina M, Post JJ, Cameron BA, et al. HIV and co-infections. Immunological Reviews. 2013;254(1):114-42. | 2013 | title/abstract | Not randomized clinical trial |
| 22 | Gikonyo C, Kamuya D, Mbete B, Njuguna P, Olotu A, Bejon P, et al. Feedback of Research Findings for Vaccine Trials: Experiences from Two Malaria Vaccine Trials Involving Healthy Children on the Kenyan Coast. Developing World Bioethics. 2013;13(1):48-56. | 2013 | title/abstract | Not randomized clinical trial |
| 23 | Mata E, Salvador A, Igartua M, Hernández RM, Pedraz JL. Malaria Vaccine Adjuvants: Latest Update and Challenges in Preclinical and Clinical Research. BioMed Research International. 2013;2013(1):282913. | 2013 | title/abstract | Not randomized clinical trial |
| 24 | Leroux-Roels G, Leroux-Roels I, Clement F, Ofori-Anyinam O, Lievens M, Jongert E, et al. Evaluation of the immune response to RTS,S/AS01 and RTS,S/AS02 adjuvanted vaccines: randomized, double-blind study in malaria-naïve adults. Hum Vaccin Immunother. 2014;10(8):2211-9. | 2014 | title/abstract | no 5-17 months age group |
| 25 | Umeh R, Oguche S, Oguonu T, Pitmang S, Shu E, Onyia JT, et al. Immunogenicity and safety of the candidate RTS,S/AS01 vaccine in young Nigerian children: A randomized, double-blind, lot-to-lot consistency trial. Vaccine. 2014;32(48):6556-62. | 2014 | full text | No SAE reported |
| 26 | Systemic Infections. ClinMicroNow. p. 1-68. | 2014 | title/abstract | Not randomized clinical trial |
| 27 | Delany I, Rappuoli R, De Gregorio E. Vaccines for the 21st century. EMBO Molecular Medicine. 2014;6(6):708-20. | 2014 | title/abstract | Not randomized clinical trial |
| 28 | Fotie J. The Potential of Peptides and Depsipeptides from Terrestrial and Marine Organisms in the Fight against Human Protozoan Diseases. Bioactive Natural Products2014. p. 279-320. | 2014 | title/abstract | Not randomized clinical trial |
| 29 | Kraft JC, Freeling JP, Wang Z, Ho RJY. Emerging Research and Clinical Development Trends of Liposome and Lipid Nanoparticle Drug Delivery Systems. Journal of Pharmaceutical Sciences. 2014;103(1):29-52. | 2014 | title/abstract | Not randomized clinical trial |
| 30 | Nankabirwa J, Brooker SJ, Clarke SE, Fernando D, Gitonga CW, Schellenberg D, Greenwood B. Malaria in school-age children in Africa: an increasingly important challenge. Tropical Medicine & International Health. 2014;19(11):1294-309. | 2014 | title/abstract | Not randomized clinical trial |
| 31 | Kaslow DC, Biernaux S. RTS,S: Toward a first landmark on the Malaria Vaccine Technology Roadmap. Vaccine. 2015;33(52):7425-32. | 2015 | title/abstract | Not randomized clinical trial |
| 32 | Teneza-Mora N, Lumsden J, Villasante E. A malaria vaccine for travelers and military personnel: Requirements and top candidates. Vaccine. 2015;33(52):7551-8. | 2015 | title/abstract | Not randomized clinical trial |
| 33 | Agnandji ST, Fernandes JF, Bache EB, Ramharter M. Clinical development of RTS,S/AS malaria vaccine: a systematic review of clinical Phase I-III trials. Future Microbiology. 2015;10(10):1553-78. | 2015 | title/abstract | Not randomized clinical trial |
| 34 | Hoffman SL, Vekemans J, Richie TL, Duffy PE. The march toward malaria vaccines. Vaccine. 2015;33:D13-D23. | 2015 | title/abstract | Not randomized clinical trial |
| 35 | Neafsey DE, Juraska M, Bedford T, Benkeser D, Valim C, Griggs A, et al. Genetic Diversity and Protective Efficacy of the RTS, S/AS01 Malaria Vaccine. New England Journal of Medicine. 2015;373(21):2025-37. | 2015 | title/abstract | Not randomized clinical trial |
| 36 | Plenary Sessions. Tropical Medicine & International Health. 2015;20(S1):1-147. | 2015 | title/abstract | Not randomized clinical trial |
| 37 | Poster Sessions. Tropical Medicine & International Health. 2015;20(S1):171-441. | 2015 | title/abstract | Not randomized clinical trial |
| 38 | Moyle PM. Progress in Vaccine Development. Current Protocols in Microbiology. 2015;36(1):18.1.1-.1.26. | 2015 | title/abstract | Not randomized clinical trial |
| 39 | Siu E, Ploss A. Modeling malaria in humanized mice: opportunities and challenges. Annals of the New York Academy of Sciences. 2015;1342(1):29-36. | 2015 | title/abstract | Not randomized clinical trial |
| 40 | Gessner BD, Wraith DC, Finn A. CNS infection safety signal of RTS,S/AS01 and possible association with rabies vaccine. Lancet (London, England). 2016;387(10026):1376. | 2016 | title/abstract | Not randomized clinical trial |
| 41 | Regules JA, Cicatelli SB, Bennett JW, Paolino KM, Twomey PS, Moon JE, et al. Fractional Third and Fourth Dose of RTS,S/AS01 Malaria Candidate Vaccine: A Phase 2a Controlled Human Malaria Parasite Infection and Immunogenicity Study. J Infect Dis. 2016;214(5):762-71. | 2016 | title/abstract | no 5-17 months age group |
| 42 | Rampling T, Ewer KJ, Bowyer G, Bliss CM, Edwards NJ, Wright D, et al. Safety and High Level Efficacy of the Combination Malaria Vaccine Regimen of RTS,S/AS01<sub>B</sub> With Chimpanzee Adenovirus 63 and Modified Vaccinia Ankara Vectored Vaccines Expressing ME-TRAP. Journal of Infectious Diseases. 2016;214(5):772-81. | 2016 | title/abstract | no 5-17 months age group |
| 43 | Burrows JN, Wells TNC. Development of medicines for the control and elimination of malaria. Advances in Malaria Research2016. p. 353-82. | 2016 | title/abstract | Not randomized clinical trial |
| 44 | Theel ES, Pritt BS. Parasites. Diagnostic Microbiology of the Immunocompromised Host2016. p. 411-66. | 2016 | title/abstract | Not randomized clinical trial |
| 45 | von Seidlein L, Hanboonkunupakarn B, Jittmala P, Pukrittayakamee S. RTS,S/AS01, a vaccine targeting pre-erythrocytic stages of Plasmodium falciparum. Emerg Top Life Sci. 2017;1(6):533-7. | 2017 | title/abstract | Not randomized clinical trial |
| 46 | Gessner BD, Knobel DL, Conan A, Finn A. Could the RTS,S/AS01 meningitis safety signal really be a protective effect of rabies vaccine? Vaccine. 2017;35(5):716-21. | 2017 | title/abstract | Not randomized clinical trial |
| 47 | Matuschewski K. Vaccines against malaria—still a long way to go. The FEBS Journal. 2017;284(16):2560-8. | 2017 | title/abstract | Not randomized clinical trial |
| 48 | Owusu-Ofori A, Owusu-Ofori S, Bates I. Global challenges of malaria risk – perspectives from transfusion-transmitted malaria. ISBT Science Series. 2017;12(1):68-72. | 2017 | title/abstract | Not randomized clinical trial |
| 49 | Tuju J, Kamuyu G, Murungi LM, Osier FHA. Vaccine candidate discovery for the next generation of malaria vaccines. Immunology. 2017;152(2):195-206. | 2017 | title/abstract | Not randomized clinical trial |
| 50 | Vandoolaeghe P, Schuerman L. The RTS,S/AS01 malaria vaccine in children 5 to 17 months of age at first vaccination. Expert Rev Vaccines. 2016;15(12):1481-93. | 2018 | title/abstract | Not randomized clinical trial |
| 51 | Valéa I, Adjei S, Usuf E, Traore O, Ansong D, Tinto H, et al. Immune response to the hepatitis B antigen in the RTS,S/AS01 malaria vaccine, and co-administration with pneumococcal conjugate and rotavirus vaccines in African children: A randomized controlled trial. Hum Vaccin Immunother. 2018;14(6):1489-500. | 2018 | title/abstract | no 5-17 months age group |
| 52 | Witte D, Cunliffe NA, Turner AM, Ngulube E, Ofori-Anyinam O, Vekemans J, et al. Safety and Immunogenicity of Seven Dosing Regimens of the Candidate RTS, S/AS01<sub>E</sub> Malaria Vaccine Integrated Within an Expanded Program on Immunization Regimen <i>A Phase II</i>, <i>Single</i>-<i>Center</i>, <i>Open</i>, <i>Controlled Trial in Infants in Malawi</i>. Pediatric Infectious Disease Journal. 2018;37(5):483-91. | 2018 | title/abstract | no 5-17 months age group |
| 53 | Kesik-Brodacka M. Progress in biopharmaceutical development. Biotechnology and Applied Biochemistry. 2018;65(3):306-22. | 2018 | title/abstract | Not randomized clinical trial |
| 54 | Muñoz-Wolf N, Lavelle EC. A Guide to IL-1 family cytokines in adjuvanticity. The FEBS Journal. 2018;285(13):2377-401. | 2018 | title/abstract | Not randomized clinical trial |
| 55 | Ndaya-Oloo P, Pitisuttithum P, Tornieporth NG, Desgrandchamps D, Munoz FM, Kochhar S, et al. Vaccine Update: Recent Progress With Novel Vaccines, and New Approaches to Safety Monitoring and Vaccine Shortage. The Journal of Clinical Pharmacology. 2018;58(S10):S123-S39. | 2018 | title/abstract | Not randomized clinical trial |
| 56 | Schwertz G, Witschel MC, Rottmann M, Leartsakulpanich U, Chitnumsub P, Jaruwat A, et al. Potent Inhibitors of Plasmodial Serine Hydroxymethyltransferase (SHMT) Featuring a Spirocyclic Scaffold. ChemMedChem. 2018;13(9):931-43. | 2018 | title/abstract | Not randomized clinical trial |
| 57 | Ward CL, Shaw D, Anane-Sarpong E, Sankoh O, Tanner M, Elger B. Defining Health Research for Development: The perspective of stakeholders from an international health research partnership in Ghana and Tanzania. Developing World Bioethics. 2018;18(4):331-40. | 2018 | title/abstract | Not randomized clinical trial |
| 58 | Lacaille-Dubois MA. Updated insights into the mechanism of action and clinical profile of the immunoadjuvant QS-21: A review. Phytomedicine : international journal of phytotherapy and phytopharmacology. 2019;60:152905. | 2019 | title/abstract | Not randomized clinical trial |
| 59 | Cawlfield A, Genito CJ, Beck Z, Bergmann-Leitner ES, Bitzer AA, Soto K, et al. Safety, toxicity and immunogenicity of a malaria vaccine based on the circumsporozoite protein (FMP013) with the adjuvant army liposome formulation containing QS21 (ALFQ). Vaccine. 2019;37(29):3793-803. | 2019 | title/abstract | Not randomized clinical trial |
| 60 | Tinto HD, Otieno W, Gesase S, Sorgho H, Otieno L, Liheluka E, et al. Long-term incidence of severe malaria following RTS, S/AS01 vaccination in children and infants in Africa: an open-label 3-year extension study of a phase 3 randomised controlled trial. Lancet Infectious Diseases. 2019;19(8):821-32. | 2019 | title/abstract | no 5-17 months age group |
| 61 | Amelia F, Iyori M, Emran TB, Yamamoto DS, Genshi K, Otsuka H, et al. Down-selecting circumsporozoite protein-based malaria vaccine: A comparison of malaria sporozoite challenge model. Parasite Immunology. 2019;41(5):e12624. | 2019 | title/abstract | Not randomized clinical trial |
| 62 | Callies DE. The ethical landscape of gene drive research. Bioethics. 2019;33(9):1091-7. | 2019 | title/abstract | Not randomized clinical trial |
| 63 | Caminade C, McIntyre KM, Jones AE. Impact of recent and future climate change on vector-borne diseases. Annals of the New York Academy of Sciences. 2019;1436(1):157-73. | 2019 | title/abstract | Not randomized clinical trial |
| 64 | Feng X, Xu W, Li Z, Song W, Ding J, Chen X. Immunomodulatory Nanosystems. Advanced Science. 2019;6(17):1900101. | 2019 | title/abstract | Not randomized clinical trial |
| 65 | Roberts DJ, Pain A, Chitnis CE. Molecular pathogenesis of malaria. Molecular Hematology2019. p. 193-206. | 2019 | title/abstract | Not randomized clinical trial |
| 66 | Szuster-Ciesielska A, Wawiórka L, Krokowski D, Grankowski N, Jarosz Ł, Lisiecka U, Tchórzewski M. Immunogenic Evaluation of Ribosomal P-Protein Antigen P0, P1, and P2 and Pentameric Protein Complex P0-(P1-P2)2 of Plasmodium falciparum in a Mouse Model. Journal of Immunology Research. 2019;2019(1):9264217. | 2019 | title/abstract | Not randomized clinical trial |
| 67 | Wallis J, Shenton DP, Carlisle RC. Novel approaches for the design, delivery and administration of vaccine technologies. Clinical & Experimental Immunology. 2019;196(2):189-204. | 2019 | title/abstract | Not randomized clinical trial |
| 68 | Williamson ED, Westlake GE. Vaccines for emerging pathogens: prospects for licensure. Clinical & Experimental Immunology. 2019;198(2):170-83. | 2019 | title/abstract | Not randomized clinical trial |
| 69 | Zheng J, Pan H, Gu Y, Zuo X, Ran N, Yuan Y, et al. Prospects for Malaria Vaccines: Pre-Erythrocytic Stages, Blood Stages, and Transmission-Blocking Stages. BioMed Research International. 2019;2019(1):9751471. | 2019 | title/abstract | Not randomized clinical trial |
| 70 | Asante KP, Ansong D, Kaali S, Adjei S, Lievens M, Nana Badu L, et al. Immunogenicity and safety of the RTS,S/AS01 malaria vaccine co-administered with measles, rubella and yellow fever vaccines in Ghanaian children: A phase IIIb, multi-center, non-inferiority, randomized, open, controlled trial. Vaccine. 2020;38(18):3411-21. | 2020 | title/abstract | no 5-17 months age group |
| 71 | Moon JE, Ockenhouse C, Regules JA, Vekemans J, Lee C, Chuang I, et al. A Phase IIa Controlled Human Malaria Infection and Immunogenicity Study of RTS,S/AS01E and RTS,S/AS01B Delayed Fractional Dose Regimens in Malaria-Naive Adults. J Infect Dis. 2020;222(10):1681-91. | 2020 | title/abstract | no 5-17 months age group |
| 72 | Rts SEEPIMALSGTRTSSEEPIMAL, Adeniji E, Asante KP, Boahen O, Compaoré G, Coulibaly B, et al. Estimating Annual Fluctuations in Malaria Transmission Intensity and in the Use of Malaria Control Interventions in Five Sub-Saharan African Countries. Am J Trop Med Hyg. 2020;103(5):1883-92. | 2020 | title/abstract | Not randomized clinical trial |
| 73 | Valéa I, Adjei S, Usuf E, Traore O, Ansong D, Tinto H, et al. Long-term immunogenicity and immune memory response to the hepatitis B antigen in the RTS,S/AS01(E) malaria vaccine in African children: a randomized trial. Hum Vaccin Immunother. 2020;16(6):1464-70. | 2020 | title/abstract | no 5-17 months age group |
| 74 | von Seidlein L, Hanboonkunupakarn B, Jittamala P, Pongsuwan P, Chotivanich K, Tarning J, et al. Combining antimalarial drugs and vaccine for malaria elimination campaigns: a randomized safety and immunogenicity trial of RTS,S/AS01 administered with dihydroartemisinin, piperaquine, and primaquine in healthy Thai adult volunteers. Hum Vaccin Immunother. 2020;16(1):33-41. | 2020 | title/abstract | no 5-17 months age group |
| 75 | Adeniji E, Asante KP, Boahen O, Compaoré G, Coulibaly B, Kaali S, et al. Estimating Annual Fluctuations in Malaria Transmission Intensity and in the Use of Malaria Control Interventions in Five Sub-Saharan African Countries. American Journal of Tropical Medicine and Hygiene. 2020;103(5):1883-92. | 2020 | title/abstract | Not randomized clinical trial |
| 76 | Ashley EA, Poespoprodjo JR. Treatment and prevention of malaria in children. Lancet Child & Adolescent Health. 2020;4(10):775-89. | 2020 | title/abstract | Not randomized clinical trial |
| 77 | Hogan AB, Winskill P, Ghani AC. Estimated impact of RTS,S/AS01 malaria vaccine allocation strategies in sub-Saharan Africa: A modelling study. PLoS medicine. 2020;17(11). | 2020 | title/abstract | Not randomized clinical trial |
| 78 | Aljarba NH, Al-Anazi MR, Shafeai MI, Rudiny FH, Bin Dajem SM, Alothaid H, et al. Interleukin-22 Polymorphisms in Plasmodium falciparum-Infected Malaria Patients. Mediators of Inflammation. 2020;2020(1):5193723. | 2020 | title/abstract | Not randomized clinical trial |
| 79 | Feeney ME. The immune response to malaria in utero. Immunological Reviews. 2020;293(1):216-29. | 2020 | title/abstract | Not randomized clinical trial |
| 80 | Gitta B, Kilian N. Diagnosis of Malaria Parasites Plasmodium spp. in Endemic Areas: Current Strategies for an Ancient Disease. BioEssays. 2020;42(1):1900138. | 2020 | title/abstract | Not randomized clinical trial |
| 81 | Greener M. Defeating malaria: new weapons against one of our deadliest foes. Prescriber. 2020;31(7-8):18-22. | 2020 | title/abstract | Not randomized clinical trial |
| 82 | Kilian N, Zhang Y, LaMonica L, Hooker G, Toomre D, Mamoun CB, Ernst AM. Palmitoylated Proteins in Plasmodium falciparum-Infected Erythrocytes: Investigation with Click Chemistry and Metabolic Labeling. BioEssays. 2020;42(6):1900145. | 2020 | title/abstract | Not randomized clinical trial |
| 83 | Kumar R, Loughland JR, Ng SS, Boyle MJ, Engwerda CR. The regulation of CD4+ T cells during malaria. Immunological Reviews. 2020;293(1):70-87. | 2020 | title/abstract | Not randomized clinical trial |
| 84 | Loiseau C, Cooper MM, Doolan DL. Deciphering host immunity to malaria using systems immunology. Immunological Reviews. 2020;293(1):115-43. | 2020 | title/abstract | Not randomized clinical trial |
| 85 | Murphy SC, Duenas DM, Richie TL, Shah SK. Reexamining the categorical exclusion of pediatric participants from controlled human infection trials. Bioethics. 2020;34(8):785-96. | 2020 | title/abstract | Not randomized clinical trial |
| 86 | Nureye D, Assefa S. Old and Recent Advances in Life Cycle, Pathogenesis, Diagnosis, Prevention, and Treatment of Malaria Including Perspectives in Ethiopia. The Scientific World Journal. 2020;2020(1):1295381. | 2020 | title/abstract | Not randomized clinical trial |
| 87 | Pérez-Mazliah D, Ndungu FM, Aye R, Langhorne J. B-cell memory in malaria: Myths and realities. Immunological Reviews. 2020;293(1):57-69. | 2020 | title/abstract | Not randomized clinical trial |
| 88 | Yap XZ, McCall MBB, Sauerwein RW. Fast and fierce versus slow and smooth: Heterogeneity in immune responses to Plasmodium in the controlled human malaria infection model. Immunological Reviews. 2020;293(1):253-69. | 2020 | title/abstract | Not randomized clinical trial |
| 89 | Datoo MS, Natama MH, Somé A, Traoré O, Rouamba T, Bellamy D, et al. Efficacy of a low-dose candidate malaria vaccine, R21 in adjuvant Matrix-M, with seasonal administration to children in Burkina Faso: a randomised controlled trial. Lancet (London, England). 2021;397(10287):1809-18. | 2021 | title/abstract | no RTS,S/AS01 vaccine |
| 90 | Lozano JM, Rodríguez Parra Z, Hernández-Martínez S, Yasnot-Acosta MF, Rojas AP, Marín-Waldo LS, Rincón JE. The Search of a Malaria Vaccine: The Time for Modified Immuno-Potentiating Probes. Vaccines (Basel). 2021;9(2). | 2021 | title/abstract | Not randomized clinical trial |
| 91 | Agyapong PD, Akite EJ, Ansah NA, Ansah PO, Asante KP, Awuni DA, et al. Baseline incidence of meningitis, malaria, mortality and other health outcomes in infants and young sub-Saharan African children prior to the introduction of the RTS,S/AS01<sub>E</sub> malaria vaccine. Malaria Journal. 2021;20(1). | 2021 | title/abstract | Not randomized clinical trial |
| 92 | Greenwood B, Cairns M, Chaponda M, Chico RM, Dicko A, Ouedraogo JB, et al. Combining malaria vaccination with chemoprevention: a promising new approach to malaria control. Malaria Journal. 2021;20(1). | 2021 | title/abstract | Not randomized clinical trial |
| 93 | Moon JE, Greenleaf ME, Regules JA, Debois M, Duncan EH, Sedegah M, et al. A phase IIA extension study evaluating the effect of booster vaccination with a fractional dose of RTS,S/AS01<sub>E</sub> in a controlled human malaria infection challenge. Vaccine. 2021;39(43):6398-406. | 2021 | title/abstract | Not randomized clinical trial |
| 94 | Abuga KM, Jones-Warner W, Hafalla JCR. Immune responses to malaria pre-erythrocytic stages: Implications for vaccine development. Parasite Immunology. 2021;43(2):e12795. | 2021 | title/abstract | Not randomized clinical trial |
| 95 | Amelo W, Makonnen E. Efforts Made to Eliminate Drug-Resistant Malaria and Its Challenges. BioMed Research International. 2021;2021(1):5539544. | 2021 | title/abstract | Not randomized clinical trial |
| 96 | Butkovich N, Li E, Ramirez A, Burkhardt AM, Wang S-W. Advancements in protein nanoparticle vaccine platforms to combat infectious disease. WIREs Nanomedicine and Nanobiotechnology. 2021;13(3):e1681. | 2021 | title/abstract | Not randomized clinical trial |
| 97 | Genito CJ, Batty CJ, Bachelder EM, Ainslie KM. Considerations for Size, Surface Charge, Polymer Degradation, Co-Delivery, and Manufacturability in the Development of Polymeric Particle Vaccines for Infectious Diseases. Advanced NanoBiomed Research. 2021;1(3):2000041. | 2021 | title/abstract | Not randomized clinical trial |
| 98 | He W, Baysal C, Lobato Gómez M, Huang X, Alvarez D, Zhu C, et al. Contributions of the international plant science community to the fight against infectious diseases in humans—part 2: Affordable drugs in edible plants for endemic and re-emerging diseases. Plant Biotechnology Journal. 2021;19(10):1921-36. | 2021 | title/abstract | Not randomized clinical trial |
| 99 | Kim E, Lim E-K, Park G, Park C, Lim J-W, Lee H, et al. Advanced Nanomaterials for Preparedness Against (Re-)Emerging Viral Diseases. Advanced Materials. 2021;33(47):2005927. | 2021 | title/abstract | Not randomized clinical trial |
| 100 | Rudrapal M, Chetia D. Malaria and Recent Developments in Antimalarial Drugs. Neglected Tropical Diseases and Phytochemicals in Drug Discovery2021. p. 499-542. | 2021 | title/abstract | Not randomized clinical trial |
| 101 | Gao Q. [Field evaluation and future applications of the world's first malaria vaccine]. Zhongguo Xue Xi Chong Bing Fang Zhi Za Zhi. 2022;33(6):551-2. | 2022 | title/abstract | Not randomized clinical trial |
| 102 | Syed YY. RTS,S/AS01 malaria vaccine (Mosquirix(®)): a profile of its use. Drugs Ther Perspect. 2022;38(9):373-81. | 2022 | title/abstract | Not randomized clinical trial |
| 103 | Björkman A, Benn CS, Aaby P, Schapira A. RTS,S/AS01 malaria vaccine-proven safe and effective? The Lancet Infectious diseases. 2023;23(8):e318-e22. | 2022 | title/abstract | Not randomized clinical trial |
| 104 | Grant J, Sagara I, Zongo I, Cairns M, Yerbanga RS, Diarra M, et al. Impact of seasonal RTS,S/AS01<sub>E</sub> vaccination plus seasonal malaria chemoprevention on the nutritional status of children in Burkina Faso and Mali. Malaria Journal. 2022;21(1). | 2022 | title/abstract | Not randomized clinical trial |
| 105 | Hutter JN, Robben PM, Lee CSE, Hamer M, Moon JE, Merino K, et al. First-in-human assessment of safety and immunogenicity of low and high doses of <i>Plasmodium falciparum</i> malaria protein 013 (FMP013) administered intramuscularly with ALFQ adjuvant in healthy malaria-naive adults. Vaccine. 2022;40(40):5781-90. | 2022 | title/abstract | no 5-17 months age group |
| 106 | Praet N, Asante KP, Bozonnat MC, Akité EJ, Ansah PO, Baril L, et al. Assessing the safety, impact and effectiveness of RTS,S/AS01<sub>E</sub> malaria vaccine following its introduction in three sub-Saharan African countries: methodological approaches and study set-up. Malaria Journal. 2022;21(1). | 2022 | title/abstract | Not randomized clinical trial |
| 107 | Thompson HA, Hogan AB, Walker PGT, Winskill P, Zongo I, Sagara I, et al. Seasonal use case for the RTS,S/AS01 malaria vaccine: a mathematical modelling study. Lancet Global Health. 2022;10(12):E1782-E92. | 2022 | title/abstract | Not randomized clinical trial |
| 108 | Akide Ndunge OB, Kilian N, Salman MM. Cerebral Malaria and Neuronal Implications of Plasmodium Falciparum Infection: From Mechanisms to Advanced Models. Advanced Science. 2022;9(36):2202944. | 2022 | title/abstract | Not randomized clinical trial |
| 109 | Kannan D, Kaur A, Salunke DB, Singh S. Toll-Like Receptor-Based Adjuvants: A Gateway Toward Improved Malaria Vaccination. Drug Development for Malaria2022. p. 319-51. | 2022 | title/abstract | Not randomized clinical trial |
| 110 | Kaur D, Sinha S, Sehgal R. Global scenario of Plasmodium vivax occurrence and resistance pattern. Journal of Basic Microbiology. 2022;62(12):1417-28. | 2022 | title/abstract | Not randomized clinical trial |
| 111 | Mahanta SK. Recent Advances in Malaria Vaccine Development. Drug Development for Malaria2022. p. 303-18. | 2022 | title/abstract | Not randomized clinical trial |
| 112 | Parikh K, Kapadia R, Pai R, Prajapati M, Shevalkar G. Emerging Formulation Technologies Against Malaria Resurgence. Drug Development for Malaria2022. p. 45-82. | 2022 | title/abstract | Not randomized clinical trial |
| 113 | Ramani K. Scientific Challenges and Treatment Opportunities in the Face of Shifting Malaria Epidemiology. Drug Development for Malaria2022. p. 25-44. | 2022 | title/abstract | Not randomized clinical trial |
| 114 | Reddy BM. Nanomedicine and Nanovaccinology Tools in Targeted Drug Delivery. Nanovaccinology as Targeted Therapeutics2022. p. 21-52. | 2022 | title/abstract | Not randomized clinical trial |
| 115 | Siddiqui A, Adnan A, Abbas M, Taseen S, Ochani S, Essar MY. Revival of the heterologous prime-boost technique in COVID-19: An outlook from the history of outbreaks. Health Science Reports. 2022;5(2):e531. | 2022 | title/abstract | Not randomized clinical trial |
| 116 | Yang G, Balzer LB, Benkeser D. Causal inference methods for vaccine sieve analysis with effect modification. Statistics in Medicine. 2022;41(8):1513-24. | 2022 | title/abstract | Not randomized clinical trial |
| 117 | Mumtaz H, Nadeem A, Bilal W, Ansar F, Saleem S, Khan QA, et al. Acceptance, availability, and feasibility of RTS, S/AS01 malaria vaccine: A review. Immunity, inflammation and disease. 2023;11(6):e899. | 2023 | title/abstract | Not randomized clinical trial |
| 118 | Sang S, Datoo MS, Otieno E, Muiruri C, Bellamy D, Gathuri E, et al. Safety and immunogenicity of varied doses of R21/Matrix-M™ vaccine at three years follow-up: A phase 1b age de-escalation, dose-escalation trial in adults, children, and infants in Kilifi-Kenya. Wellcome Open Res. 2023;8:450. | 2023 | title/abstract | no RTS,S/AS01 vaccine |
| 119 | Wiltz P. Identifying and Managing Vector-Borne Diseases in Migrants and Recent Travelers in the Emergency Department. Curr Emerg Hosp Med Rep. 2023;11(2):58-65. | 2023 | title/abstract | Not randomized clinical trial |
| 120 | Yihunie W, Kebede B, Tegegne BA, Getachew M, Abebe D, Aschale Y, et al. Systematic Review of Safety of RTS,S with AS01 and AS02 Adjuvant Systems Using Data from Randomized Controlled Trials in Infants, Children, and Adults. Clinical pharmacology : advances and applications. 2023;15:21-32. | 2023 | title/abstract | Not randomized clinical trial |
| 121 | Ajayi MY, Emeto DC. Awareness and acceptability of malaria vaccine among caregivers of under-5 children in Northern Nigeria. Malaria Journal. 2023;22(1). | 2023 | title/abstract | no 5-17 months age group |
| 122 | Borkens Y. Malaria & mRNA Vaccines: A Possible Salvation from One of the Most Relevant Infectious Diseases of the Global South. Acta Parasitologica. 2023;68(4):916-28. | 2023 | title/abstract | Not randomized clinical trial |
| 123 | Hoyt J, Okello G, Bange T, Kariuki S, Jalloh MF, Webster J, Hill J. RTS,S/AS01 malaria vaccine pilot implementation in western Kenya: a qualitative longitudinal study to understand immunisation barriers and optimise uptake. Bmc Public Health. 2023;23(1). | 2023 | title/abstract | Not randomized clinical trial |
| 124 | Merle CS, grp RSw. Implementation strategies for the introduction of the RTS,S/AS01 (RTS,S) malaria vaccine in countries with areas of highly seasonal transmission: workshop meeting report. Malaria Journal. 2023;22(1). | 2023 | title/abstract | Not randomized clinical trial |
| 125 | Price J, Gurley N, Gyapong M, Ansah EK, Awusabo-Asare K, Gyasi SF, et al. Acceptance of and Adherence to a Four-Dose RTS,S/AS01 Schedule: Findings from a Longitudinal Qualitative Evaluation Study for the Malaria Vaccine Implementation Programme. Vaccines. 2023;11(12). | 2023 | title/abstract | Not randomized clinical trial |
| 126 | Abstracts. Tropical Medicine & International Health. 2023;28(S1):3-368. | 2023 | title/abstract | Not randomized clinical trial |
| 127 | Armstrong JF, Campo B, Alexander SPH, Arendse LB, Cheng X, Davenport AP, et al. Advances in malaria pharmacology and the online guide to MALARIA PHARMACOLOGY: IUPHAR review 38. British Journal of Pharmacology. 2023;180(15):1899-929. | 2023 | title/abstract | Not randomized clinical trial |
| 128 | Bekić V, Kilian N. Novel secretory organelles of parasite origin - at the center of host-parasite interaction. BioEssays. 2023;45(9):2200241. | 2023 | title/abstract | Not randomized clinical trial |
| 129 | Cable J, Graham BS, Koup RA, Seder RA, Karikó K, Pardi N, et al. Progress in vaccine development for infectious diseases—a Keystone Symposia report. Annals of the New York Academy of Sciences. 2023;1524(1):65-86. | 2023 | title/abstract | Not randomized clinical trial |
| 130 | Choudhury AAK, Vinayagam S, Adhikari N, Saha A, Ghosh SK, Bhat HR, Patgiri SJ. Hybrid PABA-glutamic acid conjugated 1,3,5-triazine derivatives: Design, synthesis, and antimalarial activity screening targeting Plasmodium falciparum dihydro folate reductase enzyme. Chemical Biology & Drug Design. 2023;102(6):1336-52. | 2023 | title/abstract | Not randomized clinical trial |
| 131 | Irfan I, Shahi D, Joshi MC, Singh S, Abid M. Emerging Paradigm of Ivermectin and its Hybrids in Elimination of Malaria. Chemistry and Biological Activities of Ivermectin2023. p. 93-119. | 2023 | title/abstract | Not randomized clinical trial |
| 132 | Park J, Pho T, Champion JA. Chemical and biological conjugation strategies for the development of multivalent protein vaccine nanoparticles. Biopolymers. 2023;114(8):e23563. | 2023 | title/abstract | Not randomized clinical trial |
| 133 | Sarwal Y, Sarwal R. COVID-19 vaccination in pregnancy: Need for global pharmaco-vigilance. International Journal of Gynecology & Obstetrics. 2023;162(1):24-8. | 2023 | title/abstract | Not randomized clinical trial |
| 134 | Shimoyama A, Fukase K. Bacterial Glycolipid Lipid As and Their Potential as Adjuvants. Carbohydrate‐Based Therapeutics2023. p. 111-30. | 2023 | title/abstract | Not randomized clinical trial |
| 135 | Dicko A, Ouedraogo JB, Zongo I, Sagara I, Cairns M, Yerbanga RS, et al. Seasonal vaccination with RTS,S/AS01(E) vaccine with or without seasonal malaria chemoprevention in children up to the age of 5 years in Burkina Faso and Mali: a double-blind, randomised, controlled, phase 3 trial. The Lancet Infectious diseases. 2024;24(1):75-86. | 2024 | title/abstract | no 5-17 months age group |
| 136 | Natama HM, Salkeld J, Somé A, Soremekun S, Diallo S, Traoré O, et al. Safety and efficacy of the blood-stage malaria vaccine RH5.1/Matrix-M in Burkina Faso: interim results of a double-blind, randomised, controlled, phase 2b trial in children. The Lancet Infectious diseases. 2024. | 2024 | title/abstract | no RTS,S/AS01 vaccine |
| 137 | Plieskatt J, Ofori EA, Naghizadeh M, Miura K, Flores-Garcia Y, Borbye-Lorenzen N, et al. ProC6C, a novel multi-stage malaria vaccine, elicits functional antibodies against the minor and central repeats of the Circumsporozoite Protein in human adults. Front Immunol. 2024;15:1481829. | 2024 | title/abstract | no 5-17 months age group |
| 138 | Tajudeen YA, Oladipo HJ, Yusuff SI, Abimbola SO, Abdulkadir M, Oladunjoye IO, et al. A landscape review of malaria vaccine candidates in the pipeline. Trop Dis Travel Med Vaccines. 2024;10(1):19. | 2024 | title/abstract | Not randomized clinical trial |
| 139 | Asante KP, Mathanga DP, Milligan P, Akech S, Oduro A, Mwapasa V, et al. Feasibility, safety, and impact of the RTS,S/AS01 E malaria vaccine when implemented through national immunisation programmes: evaluation of cluster-randomised introduction of the vaccine in Ghana, Kenya, and Malawi. Lancet (London, England). 2024;403(10437):1660-70. | 2024 | title/abstract | Not randomized clinical trial |
| 140 | Upadhyay C, Bhattacharya S, Kumar S, Vashisht K, Zhang X, Gagnon D, et al. Synthesis and Evaluation of Fluorinated Piperazine-Hydroxyethylamine Analogues as Potential Antiplasmodial Candidates. ChemMedChem.n/a(n/a):e202400616. | 2024 | title/abstract | Not randomized clinical trial |
| 141 | Vujevic A, Weld ED. Malaria in pregnancy: Modern approaches—Spotlight commmentary. British Journal of Clinical Pharmacology.n/a(n/a). | 2024 | title/abstract | Not randomized clinical trial |
| 142 | News. Chemistry & Industry. 2024;88(2):5-13. | 2024 | title/abstract | Not randomized clinical trial |
| 143 | Avalos-Padilla Y, Fernàndez-Busquets X. Nanotherapeutics against malaria: A decade of advancements in experimental models. WIREs Nanomedicine and Nanobiotechnology. 2024;16(2):e1943. | 2024 | title/abstract | Not randomized clinical trial |
| 144 | Balmith M, Basson C, Brand SJ. The Malaria Burden: A South African Perspective. Journal of Tropical Medicine. 2024;2024(1):6619010. | 2024 | title/abstract | Not randomized clinical trial |
| 145 | Bragazzi NL, Watad A, Shoenfeld Y. Vaccine Adjuvants: History, Role, Mechanisms of Action, and Side Effects. Autoimmune Disorders2024. p. 15-27. | 2024 | title/abstract | Not randomized clinical trial |
| 146 | Chutiyami M. Recent Trends in Malaria Vaccine Research Globally: A Bibliometric Analysis From 2005 to 2022. Journal of Parasitology Research. 2024;2024(1):8201097. | 2024 | title/abstract | Not randomized clinical trial |
| 147 | Euliano EM, Agrawal A, Yu MH, Graf TP, Henrich EM, Kunkel AA, et al. Intra-lymph node crosslinking of antigen-bearing polymers enhances humoral immunity and dendritic cell activation. Bioengineering & Translational Medicine. 2024;9(6):e10705. | 2024 | title/abstract | Not randomized clinical trial |
| 148 | Fowkes FJ, Robinson LJ, Simpson JA, Thriemer K, Laman M, Oo WH. The challenges of malaria elimination in the Asia–Pacific region. Medical Journal of Australia. 2024;221(4):182-5. | 2024 | title/abstract | Not randomized clinical trial |
| 149 | Oduoye MO, Haider MU, Marsool MDM, Kareem MO, Adedayo AE, Abdulkarim AS, et al. Unlocking the potential of novel RTS, S/AS01, and R21/Matrix-M™ malaria vaccines in African nations. Health Science Reports. 2024;7(1):e1797. | 2024 | title/abstract | Not randomized clinical trial |
| 150 | Patel P, Bagada A, Vadia N. Epidemiology and Current Trends in Malaria. Rising Contagious Diseases2024. p. 261-82. | 2024 | title/abstract | Not randomized clinical trial |
| 151 | Skorokhod O, Vostokova E, Gilardi G. The role of P450 enzymes in malaria and other vector-borne infectious diseases. BioFactors. 2024;50(1):16-32. | 2024 | title/abstract | Not randomized clinical trial |
| 152 | Zulu MD, Msuku H, Stanley CC, Phiri VS, Topazian HM, Chinkhumba J, et al. An intervention to improve lumbar puncture rates for meningitis surveillance in children at four secondary health facilities in Malawi: A before/after analysis. Tropical Medicine & International Health. 2024;29(6):499-506. | 2024 | title/abstract | Not randomized clinical trial |
| 153 | Versteeg L, Pollet J. mRNA Vaccines for Malaria and Other Parasitic Pathogens. Trends in mRNA Vaccine Research2025. p. 303-23. | 2024 | title/abstract | Not randomized clinical trial |
| 154 | Ali MS, Stockdale L, Sagara I, Zongo I, Yerbanga RS, Mahamar A, et al. The anti-circumsporozoite antibody response to repeated, seasonal booster doses of the malaria vaccine RTS,S/AS01(E). NPJ Vaccines. 2025;10(1):26. | 2025 | title/abstract | Not randomized clinical trial |
| 155 | Liang J, Yao L, Liu Z, Chen Y, Lin Y, Tian T. Nanoparticles in Subunit Vaccines: Immunological Foundations, Categories, and Applications. Small. 2025;21(1):2407649. | 2025 | title/abstract | Not randomized clinical trial |
| 156 | Nagaprasad P, Chandu B, Panigrahi PK. Exploration of Biocompatibility and Toxicity of Nanomaterials in Diagnosis and Treatment of Infectious Diseases. Sustainable Nanomaterials for Treatment and Diagnosis of Infectious Diseases2025. p. 269-310. | 2025 | title/abstract | Not randomized clinical trial |
| 157 | Olumide Adeleke T, Aigbiremo Oboh M, Yetunde O, Agnes Akinlosotu M, Naomi Oluwasanya A, Victoria Adeleke O, Samson Timothy K. Awareness, Acceptability and Factors Influencing Malaria Vaccine Uptake Among Caregivers of Children Under 5 in South-Western Nigeria. Child: Care, Health and Development. 2025;51(1):e70029. | 2025 | title/abstract | Not randomized clinical trial |
| 158 | Yoo R, Jore MM, Julien J-P. Targeting Bottlenecks in Malaria Transmission: Antibody-Epitope Descriptions Guide the Design of Next-Generation Biomedical Interventions. Immunological Reviews. 2025;330(1):e70001. | 2025 | title/abstract | Not randomized clinical trial |
| **INCLUDED STUDIES** | | | | |
| **N°** | **Study ID** | **Year** | **Screening** | **Identification** |
| 1 | Owusu-Agyei S, Ansong D, Asante K, Kwarteng Owusu S, Owusu R, Wireko Brobby NA, et al. Randomized controlled trial of RTS,S/AS02D and RTS,S/AS01E malaria candidate vaccines given according to different schedules in Ghanaian children. PloS one. 2009;4(10):e7302. | 2009 | full text | Pubmed |
| 2 | Lusingu J, Olotu A, Leach A, Lievens M, Vekemans J, Olivier A, et al. Safety of the malaria vaccine candidate, RTS,S/AS01E in 5 to 17 month old Kenyan and Tanzanian Children. PloS one. 2010;5(11):e14090. | 2010 | full text | Pubmed |
| 3 | Agnandji ST, Lell B, Soulanoudjingar SS, Fernandes JF, Abossolo BP, Conzelmann C, et al. First results of phase 3 trial of RTS,S/AS01 malaria vaccine in African children. N Engl J Med. 2011;365(20):1863-75. | 2011 | full text | Wiley Online Library |
| 4 | Minsoko PA, Lell B, Fernandes JF, Abossolo BP, Kabwende AL, Adegnika AA, et al. Efficacy and Safety of the RTS,S/AS01 Malaria Vaccine during 18 Months after Vaccination: A Phase 3 Randomized, Controlled Trial in Children and Young Infants at 11 African Sites. PLoS medicine. 2014;11(7). | 2014 | full text | Web of Science |
| 5 | Otieno L, Oneko M, Otieno W, Abuodha J, Owino E, Odero C, et al. Safety and immunogenicity of RTS, S/AS01 malaria vaccine in infants and children with WHO stage 1 or 2 HIV disease: a randomised, double-blind, controlled trial. Lancet Infectious Diseases. 2016;16(10):1134-44. | 2016 | full text | Web of Science |
| 6 | Guerra Mendoza Y, Garric E, Leach A, Lievens M, Ofori-Anyinam O, Pirçon JY, et al. Safety profile of the RTS,S/AS01 malaria vaccine in infants and children: additional data from a phase III randomized controlled trial in sub-Saharan Africa. Hum Vaccin Immunother. 2019;15(10):2386-98. | 2019 | full text | Pubmed |
| 7 | Otieno L, Mendoza YG, Adjei S, Agbenyega T, Agnandji ST, Aide P, et al. Safety and immunogenicity of the RTS,S/AS01 malaria vaccine in infants and children identified as HIV-infected during a randomized trial in sub-Saharan Africa. Vaccine. 2020;38(4):897-906. | 2020 | full text | Web of Science |
| 8 | Samuels AM, Ansong D, Kariuki SK, Adjei S, Bollaerts A, Ockenhouse C, et al. Efficacy of RTS,S/AS01(E) malaria vaccine administered according to different full, fractional, and delayed third or early fourth dose regimens in children aged 5-17 months in Ghana and Kenya: an open-label, phase 2b, randomised controlled trial. The Lancet Infectious diseases. 2022;22(9):1329-42. | 2022 | full text | Pubmed |
| 9 | Bejon P, Lusingu J, Olotu A, Leach A, Lievens M, Vekemans J, Mshamu S, Lang T, Gould J, Dubois MC, Demoitié MA, Stallaert JF, Vansadia P, Carter T, Njuguna P, Awuondo KO, Malabeja A, Abdul O, Gesase S, Mturi N, Drakeley CJ, Savarese B, Villafana T, Ballou WR, Cohen J, Riley EM, Lemnge MM, Marsh K, von Seidlein L. Efficacy of RTS,S/AS01E vaccine against malaria in children 5 to 17 months of age. N Engl J Med. 2008 Dec 11;359(24):2521-32. doi: 10.1056/NEJMoa0807381. Epub 2008 Dec 8. PMID: 19064627; PMCID: PMC2655100. | 2009 | full text | Citation searching |
| 10 | Olotu A, Lusingu J, Leach A, Lievens M, Vekemans J, Msham S, Lang T, Gould J, Dubois MC, Jongert E, Vansadia P, Carter T, Njuguna P, Awuondo KO, Malabeja A, Abdul O, Gesase S, Mturi N, Drakeley CJ, Savarese B, Villafana T, Lapierre D, Ballou WR, Cohen J, Lemnge MM, Peshu N, Marsh K, Riley EM, von Seidlein L, Bejon P. Efficacy of RTS,S/AS01E malaria vaccine and exploratory analysis on anti-circumsporozoite antibody titres and protection in children aged 5-17 months in Kenya and Tanzania: a randomised controlled trial. Lancet Infect Dis. 2011 Feb;11(2):102-9. doi: 10.1016/S1473-3099(10)70262-0. Epub 2011 Jan 13. Erratum in: Lancet Infect Dis. 2011 Mar;11(3):159. PMID: 21237715; PMCID: PMC3341451. | 2011 | full text | Citation searching |
| 11 | Olotu A, Fegan G, Wambua J, Nyangweso G, Leach A, Lievens M, Kaslow DC, Njuguna P, Marsh K, Bejon P. Seven-Year Efficacy of RTS,S/AS01 Malaria Vaccine among Young African Children. N Engl J Med. 2016 Jun 30;374(26):2519-29. doi: 10.1056/NEJMoa1515257. PMID: 27355532; PMCID: PMC4962898 | 2016 | full text | Citation searching |
